# Editorial: RNA vaccines for prevalent and newly emerging diseases

**DOI:** 10.3389/fimmu.2024.1474260

**Published:** 2024-08-23

**Authors:** César López-Camacho, Mohamad Gabriel Alameh, Dan Peer, Pieter Culis

**Affiliations:** ^1^ Nuffield Department of Medicine, The Jenner Institute, University of Oxford, Oxford, United Kingdom; ^2^ Perelman School of Medicine, University of Pennsylvania, Philadelphia, PA, United States; ^3^ Laboratory of Precision NanoMedicine, The Shmunis School of Biomedicine and Cancer Research, Tel Aviv, Israel; ^4^ Department of Materials Sciences and Engineering, Iby and Aladar Fleischman Faculty of Engineering, Tel Aviv University, Tel Aviv, Israel; ^5^ Center for Nanoscience and Nanotechnology, Tel Aviv University, Tel Aviv, Israel; ^6^ Tel Aviv, Israel and Cancer Biology Research Center, Tel Aviv University, Tel Aviv, Israel; ^7^ Department of Biochemistry and Molecular Biology, University of British Columbia, Vancouver, BC, Canada

**Keywords:** vaccines and antiviral, RNA, mRNA, mRNA vaccination, emerging pathogens

## Introduction

Faced with rising threats from both common and new infectious diseases, RNA vaccines have proven to be a game-changing technology, showing exceptional efficacy and flexibility. Their rapid deployment during the COVID-19 pandemic underscores their transformative potential in disease prevention and management. This Research Topic compiles significant contributions exploring RNA vaccines’ capabilities and challenges across various infectious agents and immune responses. The editorial summarizes key insights from original research and review articles, covering topics from novel RNA vaccines for parasitic diseases like malaria to long-term immune response evaluations and advanced delivery systems. Each study highlights recent advancements in RNA vaccine technology and emphasizes the ongoing need for research and collaboration to tackle global health challenges. This issue, reflecting significant interest in RNA vaccines, has garnered 36,248 views and 5,602 downloads, underscoring the broad interest in advancing this technology against both common and emerging diseases.

This Research Topic includes original manuscripts together with perspectives and reviews.

## Original manuscripts

**Malaria Vaccine Development:** In the study by Johnson et al., a novel vaccine targeting PfGBP130, a protein crucial for *Plasmodium falciparum*’s invasion of red blood cells, is explored. This blood stage of malaria is critical as it triggers disease symptoms. The research demonstrates that the vaccine elicits a strong immune response, significantly inhibiting parasite invasion, and presents a promising route for malaria prevention. This work extends the traditional use of mRNA vaccines, known for rapid deployment against viruses, to combat parasitic diseases like malaria. This shift highlights the adaptability of mRNA vaccines and their potential in tackling diverse pathogenic threats, marking a significant step forward in the fight against various infectious diseases.

The findings contribute to ongoing efforts to develop effective blood-stage vaccines, including other candidates published elsewhere such as PfRH5, MSP3, and AMA1, which target key proteins involved in merozoite invasion of red blood cells. These vaccines are crucial for controlling malaria’s impact on health. Johnson et al.‘s study emphasizes the need to advance these candidates to clinical trials to achieve significant breakthroughs in malaria prevention and control.

**Long-term Antibody Responses:** Serwanga et al. conducted a comprehensive study evaluating long-term antibody responses to Moderna’s mRNA-1273 COVID-19 vaccine in a Sub-Saharan African cohort, often underrepresented in vaccine studies. The research tracked the persistence of Spike-IgG and Spike-IgA antibodies for over a year post-vaccination, revealing a robust and sustained presence, suggesting prolonged immunity. This finding implies that immediate booster doses may not be necessary, highlighting the need to tailor booster strategies to specific demographic needs, especially in regions with logistical challenges.

The study’s results contrast with findings from non-African cohorts, where antibody levels typically decline within months, leading to recommendations for booster doses. The extended antibody presence in the Sub-Saharan cohort may indicate unique immune modulation influenced by genetic diversity or previous pathogen exposure. These findings could challenge current understandings of vaccine longevity and underscore the importance of adapting vaccination strategies to different populations. Further research is needed to validate these results and refine global vaccination approaches to ensure they are effective and tailored to diverse communities.

**Rabies Vaccine Efficacy:** Li et al. conducted a ground-breaking study on an mRNA vaccine (RV021) encoding the rabies virus glycoprotein, showing significant immunogenicity and efficacy in mice. The two-dose vaccine regimen induced strong neutralizing antibodies and a potent cellular immune response lasting at least 260 days, suggesting a promising strategy for human rabies prevention. Compared to traditional inactivated vaccines, RV021 produced a more durable immune response, highlighting the broader potential of mRNA technology beyond coronaviral infections, including zoonotic diseases.

Although not covered in Li et al.‘s study, the ChAdOx2 RabG vaccine from the Jenner Institute, using a single-dose viral vector approach, has shown similar promise in Phase 1 trials, maintaining neutralizing antibodies for at least a year. This single-dose vaccine could simplify logistics and reduce costs, particularly in regions where cold chain maintenance and multiple doses are challenging. While the Oxford vaccine offers logistical benefits, RV021’s two-dose regimen underscores the robust potential of mRNA technology. Both vaccines provide valuable insights into effective rabies prevention strategies.

**Sleep and Vaccine Efficacy:** Izuhara et al. studied the effect of sleep duration on antibody responses to mRNA COVID-19 vaccination, emphasizing sleep’s crucial role in enhancing vaccine efficacy. Using objective sleep measurements, the research found that longer sleep durations, especially after booster doses, were linked to higher antibody levels. This study differentiates between subjective and objective sleep data, showing that quantifiable measurements are more reliable indicators of sleep’s impact on immunity.

The findings highlight the relationship between sleep and the immune system, which is regulated by circadian rhythms. Disruptions in these rhythms can impact cytokine production and immune cell activity, potentially affecting vaccine response. Aligning vaccination with optimal circadian phases could improve vaccine-induced immunity, presenting a novel approach for public health strategies. Izuhara et al.‘s research underscores the importance of adequate sleep post-vaccination and suggests integrating circadian biology into vaccination schedules to enhance efficacy. This approach could significantly benefit public health responses during pandemics and improve overall immune readiness against infectious diseases.

**T-cell Responses in Marginalized Groups:** Gainullin et al. examined T-cell responses to mRNA COVID-19 vaccination in people who use drugs (PWUD), a group often underrepresented in clinical research. The study found that, despite potential immune suppression from substance use, PWUD developed robust T-cell responses post-vaccination, similar to non-using groups. Pre-vaccination, there was no increased T-cell cross-reactivity to SARS-CoV-2 in PWUD compared to controls, indicating the observed immune responses were due to the vaccine. Post-vaccination, both CD4+ and CD8+ T-cell responses in PWUD were polyfunctional, producing various protective cytokines crucial for a comprehensive antiviral response. This highlights that standard mRNA vaccines effectively stimulate appropriate immune responses in PWUD.

The findings stress the importance of including marginalized groups in vaccination campaigns, as their immune response to mRNA vaccines is comparable to the general population. This research advocates for public health strategies to ensure vaccines are accessible to all, especially those at higher risk due to lifestyle or health conditions.

**Research Trends in RNA Vaccines:** Chen et al. performed a bibliometric analysis to examine the surge in RNA vaccine research during the COVID-19 pandemic (2020–2023). Their study highlights a peak in publications in 2022, reflecting rapid global scientific mobilization to address the pandemic with RNA vaccine advancements. This period marked significant scientific development and demonstrated the critical role of RNA vaccines in global health emergencies. The analysis identifies the United States and China as key contributors, serving as major research hubs. These countries’ central roles underscore the international nature of efforts to develop innovative vaccine technologies. Chen et al. also highlights the significant contributions from leading institutions, with Harvard University emerging as a key player in the research network. The study maps the collaborative landscape across continents, revealing how interconnected efforts have driven advancements in RNA vaccine development.

The study emphasizes the need for more equitable global collaboration. Disparities in cooperation could impede vaccine progress, highlighting the importance of fostering stronger partnerships between developed and developing countries. This approach aims to balance the distribution of research resources and initiatives, ensuring effective global responses to future pandemics.

## Perspective and reviews

**The Good, the Bad, and the Ugly of mRNA-LNP Vaccines:** Igyártó and Qin‘s review, “The mRNA-LNP Vaccines – The Good, the Bad, and the Ugly?” critically examines mRNA-LNP vaccines, particularly spotlighted during the COVID-19 pandemic. The review highlights their high efficacy and rapid deployment in controlling outbreaks but also discusses significant issues such as safety concerns and variable efficacy over time. The authors delve into potential adverse effects, including inflammatory responses triggered by lipid nanoparticles (LNPs) and contaminants such as double-stranded RNA. Additionally, they highlight the persistence of vaccine components (mRNA and spike protein) in the body longer than expected, challenging the assumption of rapid clearance and suggesting implications for long-term safety.

This review acknowledges the successes of mRNA-LNP vaccines but emphasizes the need for ongoing research to resolve these issues and ensure long-term safety and efficacy. It calls for a balanced approach to vaccine development, carefully weighing potential risks and benefits to fully harness the promise of this technology.

**RNA Sensing and Signaling:** Luan et al., in their review “Innate Immune Responses to RNA: Sensing and Signaling,” delve into the mechanisms by which cells detect and respond to RNA, highlighting the roles of sensors like RIG-I, MDA5, and TLRs in innate immunity. These RNA sensors are crucial for recognizing viral RNA and triggering immune responses, critical for the efficacy of RNA vaccines. The review explains how various RNA sensors and their distinct activation pathways allow cells to distinguish between self and non-self RNA, facilitating appropriate immune responses. It covers the structural and biochemical aspects of RNA sensor activation and regulation, showing how these pathways contribute to antiviral defenses and immune homeostasis. Luan et al. emphasize that understanding these interactions is key to designing RNA vaccines that trigger effective immune reactions while minimizing adverse effects.

Their work underscores the potential for optimizing RNA vaccine formulations to enhance efficacy and safety. By elucidating RNA sensor mechanisms, the review sets the stage for future research into improving RNA-based therapies, highlighting the importance of RNA sensing and signaling in developing next-generation vaccines and treatments for various infections.

**Targeted mRNA-Delivery for Improved Vaccine Design:** Clemente et al., in their review “Straight to the Point: Targeted mRNA-Delivery to Immune Cells for Improved Vaccine Design,” explore how targeting mRNA vaccines directly to dendritic cells (DCs) can enhance immunogenicity and vaccine efficiency. DCs, as key antigen-presenting cells, play a critical role in the immune response, and directing mRNA to these cells could revolutionize vaccine strategies. The review details methods for targeting mRNA to DCs, such as using specific ligands to bind DC receptors, significantly increasing the potential for a robust and targeted immune response with fewer side effects. This targeted approach could lower vaccine dosage requirements and reduce systemic inflammatory responses, optimizing both efficacy and safety. Clemente et al. emphasize the transformative potential of targeted mRNA delivery for future vaccine development, suggesting significant advancements in precision immunization against various diseases. Focusing on dendritic cells allows researchers to harness the immune system more efficiently, paving the way for precise and effective vaccines.

The concept of targeting immune cells such as DCs has been explored with viral vectors, such as adenoviruses, which are highly efficient in gene delivery and trigger strong immune responses. However, adenoviruses pose risks, including pre-existing immunity and potential inflammation. In contrast, mRNA vaccines offer a flexible, safer alternative, avoiding genome integration and allowing rapid production and modification. Clemente et al.‘s review highlights mRNA technology’s potential for high-precision targeted delivery, suggesting it could revolutionize vaccine development and provide safer, more effective solutions for both existing and emerging diseases.

## Discussion and challenges ahead

The research in this Research Topic of *Frontiers in Immunology* underscores the transformative potential of RNA vaccines in tackling a wide range of diseases, from *Plasmodium falciparum* (malaria) and the rabies virus to SARS-CoV-2. These studies highlight the adaptability of RNA vaccines and their significant global health impact. Reflecting on challenges for the near future:

1. **Promoting Global Collaboration and Access:** Emphasize the need for enhanced global collaboration and equitable distribution of research resources. Ensuring RNA vaccine access worldwide, especially in developing countries, requires stronger international partnerships and addressing disparities in research funding. This is crucial for effective responses to future pandemics and equitable health outcomes globally.
2. **Achieving Single-Dose Efficacy:** Developing single-dose RNA vaccines is a major future challenge. Single-dose vaccines would streamline logistics, reduce costs, and improve compliance, particularly in resource-limited areas. Creating vaccines that offer long-lasting immunity with one dose requires innovative approaches in formulation and delivery to maximize their global impact.
3. **Integrating Circadian Biology in Vaccination:** Aligning vaccination times with optimal circadian phases could enhance immune responses and vaccine efficacy. Further research is needed to integrate the sleep factor into vaccination schedules to improve the effectiveness of vaccination programs.
4. **Ensuring Safety and Managing Long-Term Effects:** Ongoing research is essential to evaluate the safety of RNA vaccines, particularly the use of vehicle platforms and other components. Monitoring potential inflammatory responses and long-term effects, along with transparent communication with the public, is key to maintaining confidence in RNA vaccine technology.

The future of RNA vaccines is promising, with the potential to revolutionize disease prevention and treatment. This Research Topic highlights the significant progress in RNA vaccine research and the importance of continuing efforts to advance this transformative technology for global health.

